# Laparoscopic transduodenal ampullectomy: initial experience from a single center

**DOI:** 10.3389/fonc.2023.1113490

**Published:** 2023-07-13

**Authors:** Pan Gao, He Cai, Zhong Wu, Bing Peng, Yunqiang Cai

**Affiliations:** Division of Pancreatic Surgery, Department of General Surgery, West China Hospital, Sichuan University, Chengdu, China

**Keywords:** laparoscopy, function-preserving surgery, ampullectomy, pancreaticoduodenectomy, ampulla of Vater

## Abstract

**Background:**

Laparoscopic transduodenal ampullectomy (LTDA) is a function-preserving surgery for pre-malignant tumors of the ampulla of Vater (AoV). However, it is technically challenging, and only a few case reports of LTDA are available in the literature.

**Methods:**

A total of 43 cases of pre-malignant tumors of AoV were operated in West China Hospital, Sichuan University between January 2017 and July 2022. Among these patients, 9 patients (group 1) underwent LTDA, 19 patients (group 2) underwent laparoscopic pancreaticoduodenectomy (LPD), and 15 patients (group 3) underwent open transduodenal ampullectomy (OTDA). Prospective collection and retrospective analysis of the demographic characteristics, intraoperative variables, and postoperative variables were carried out.

**Results:**

The patients in the three groups were comparable in terms of sex, age, body mass index, tumor size, and preoperative blood tests. In comparison to the patients in group 2, the patients in group 1 were found to require less operative time (159.7 ± 47.5 min vs. 298.1 ± 62.6, *p* < 0.01) and suffered lower blood losses (23.3 ± 16.7 ml vs. 156.8 ± 112.1, *p* = 0.002) and complications. Moreover, the postoperative hospital stays (POHS) were significantly shorter for patients in group 1 (9.0 ± 5.3 days vs. 15.5 ± 7.3 days, *p* = 0.04). Compared to patients who underwent OTDA, the patients in LTDA suffered from less blood loss. The operative time and post-operative details were comparable.

**Conclusion:**

Therefore, LTDA was found to be safe and feasible in the setting of pre-malignant tumors of AoV in well-selected patients. However, multidisciplinary preoperative planning is essential before the surgery.

## Introduction

Tumors of the ampulla of Vater (AoV) are relatively uncommon, accounting for only 5% of all cancers of the gastrointestinal system, with a prevalence of 0.04%–0.12% in cases of autopsy (1). There has been frequent identification of ampullary tumors owing to better imaging techniques and increasing endoscopic surveillance. Strategies for treating the tumors of AoV include endoscopic papillectomy (EP), transduodenal ampullectomy (TDA), and pancreaticoduodenectomy (PD) (2). PD is considered to be the standard treatment for cancers in AoV (3). However, the high degree of invasiveness associated with PD comes into question in the setting of pre-malignant lesions of the AoV. EP is considered to be a safer alternative for treating pre-malignant lesions of the AoV. Despite having lower rates of morbidity and mortality, EP is associated with a risk of incomplete resection (4). Compared with PD, TDA is a less invasive and function-preserving surgery. It has lower rates of peri-operative morbidity and mortality than PD and has been suggested as an alternative surgical treatment for ampullary adenoma (5). Laparoscopic transduodenal ampullectomy (LTDA) is more challenging than open surgery, and only a few case reports of LTDA are available in the literature (6–8). In this single-center study, we aim to evaluate the safety and effectiveness of LTDA for the treatment of ampullary tumors.

## Materials and methods

A total of 118 cases of tumors of AoV were operated by a single surgeon (Prof. Peng) in West China Hospital, Sichuan University, Chengdu, China, between January 2017 and July 2022. Among these patients, 10 patients (group 1) underwent LTDA. One patient in group 1 converted to laparoscopic pancreaticoduodenectomy (LPD) was excluded from this study. LPD was performed on 19 patients (group 2) with benign or pre-malignant tumors of AoV. Eighty-nine cases of adenocarcinoma of AoV were excluded from this study. Fifteen cases of open transduodenal ampullectomy (OTDA) operated by other surgeons (group 3) were also included as a control group. Generally, multidisciplinary preoperative planning was routinely carried out for each patient. The final selection of the treatment modality was determined based on a consensus of the endoscope doctor, surgeon, pathologist, and patient. Surgery was considered in the case of malignant lesions, or tumors of AoV with intraductal involvement, or tumor size larger than 4 cm or other technical difficulty for endoscopic papillectomy. Each patient underwent endoscopic biopsy and endoscopic ultrasound examination preoperatively. For patients where the biopsy suggests malignancy or highly suspected malignancy, or for patients where the endoscopic ultrasound suggests intraductal involvement > 2 mm, PD was preferentially recommended. For patients with moderate to severe atypical hyperplasia and intraductal involvement > 2 mm, TDA was recommended. The decision to choose open surgery or laparoscopic surgery was a comprehensive decision made by the surgeon based on their surgical experience, technical proficiency, and a comprehensive assessment of the patient’s condition. Demographic characteristics (age, gender, BMI, and pathological diagnosis), intraoperative variables (conversion, operative time, estimated blood loss, transfusion, pancreatic texture, and diameter of the main pancreatic duct), and postoperative variables (time for oral intake, postoperative hospital stay, and complications) were prospectively collected and retrospectively analyzed. Informed consent for participation was obtained from all the patients, and this study was ethically cleared by the Ethics Committee of Sichuan University.

### Surgical techniques

#### Patient position and trocar distributions

Patients were placed in a supine position with two legs separated. The surgeon stood on the left side of the patient. The first surgeon stood on the right side of the patient. The scope assistant stood between the patient’s two legs. The observing trocar was located at the inferior umbilicus. Four trocars were distributed symmetrically at the midclavicular line and anterior axillary line.

#### The operative procedure for LTDA

The operative procedures began with a careful exploration of the entire abdominal cavity. The hepatic flexure of the colon and mesocolon was fully taken down. A wide Kocher maneuver was performed, and the duodenum and pancreatic head were fully mobilized. Suturing of the anterior wall of the descending duodenum was carried out, and it was retracted to the left abdominal wall. The location of AoV was identified using palpation or intraoperative laparoscopic ultrasonography. A 3-cm longitudinal duodenotomy was performed, and the ampullary lesion was visualized and ligated for retraction. For lifting the lesion, physiological saline with noradrenaline (5 ml) was injected into the submucosa. The duodenal mucosa was incised at least 1 cm from the tumor to ensure a negative margin. The Wirsung duct was then identified, cut sharply with care to ensure an adequate margin, and a stent was placed into it. The reconstruction of the pancreatic duct to duodenum mucosa was carried out using 4-0 absorbable sutures. The mucosal defect at the distal side of AoV was also repaired using absorbable sutures. A cephalad dissection was continued, and the common bile duct was identified and opened. A 50-cm stent was placed into the common bile duct, and the other end of the stent remained outside the abdominal cavity. The bile juice was completely drained out to prevent bile juice contamination in the abdominal and duodenal cavities. The sphincteroplasty of the common bile duct to duodenum mucosa was carried out with 4-0 absorbable sutures at the 3, 6, and 9 o’clock positions. The specimen was then removed and sent for rapid frozen pathologic examination. Suturing of the common bile duct to duodenum mucosa was performed at the 12 o’clock position. While the stent in the pancreatic duct was preserved, the stent in the common bile duct was removed. The duodenal incision was then closed transversely with two-layer sutures, and two drainages were placed around the duodenal incision.

#### The operative procedure for LPD

The patient position and trocar distributions were the same as that for LTDA. The details of operative procedure and pancreaticojejunostomy were demonstrated in our previous study (9). Briefly, four layers of duct-to-mucosa pancreaticojejunostomy with an internal stent were performed. Running suturing was performed for the outer layer using 4-0 Prolene. A figure-eight suture plus running suturing was carried out for the inner layer by using 5-0 PDS suture.

#### Peri-operative management

Gastroscopy and biopsy of the lesion were performed for all patients. Endoscopic ultrasonography (EUS) was used to observe and assess the T factor and superficial bile or pancreatic duct progression. The nasogastric tube was removed on the first postoperative day (POD). Patients began the intake of water on the first POD, and the oral intake of liquid food was started to post the first passage of flatus. Computed tomography was performed on POD 3, and the drainages were removed on POD 5.

### Statistical analyses

Statistical analyses were carried out using SPSS 22.0 for Windows (SPSS Inc., Chicago, IL, USA), and the numerical data were expressed as mean ±standard deviation. The chi-square test, or Fisher’s exact test, was used to compare the categorical variables, and the independent *t*-test, or Mann–Whitney *U*-test, was used to compare the continuous variables. A value of *p* < 0.05 was considered statistically significant.

## Results

The demographic characteristics of the patients are shown in **Table 1**. A total of 43 cases of surgery for pre-malignant tumor of AoV were included in this study, including 9 cases of LTDA, 15 cases of OTDA, and 19 cases of LPD. The median age of these patients was 62 years (range, 38 to 78 years). The age, sex, and BMI were comparable among the three groups.

**Table 1:** The demographic characteristics of patients.

Variables	LTDA	LPD	OTDA	*p* ^1^-value	*p* ^2^-value
No. of patients	9	19	15	–	
Age (years)	64.1 ± 13.2	63.1 ± 11.3	61.6 ± 9.5	0.76	0.34
Sex (F/M)	3/6	6/13	7/8	0.93	0.68
BMI (kg/m^2^)	22.4 ± 2.4	21.6 ± 2.3	23.6 ± 1.8	0.66	0.78
Diagnosis				0.89	0.85
TVA	3	5	6		
Villous adenoma	2	3	2		
Tubular adenoma	1	2	2		
CIHGIN	1	1	2		
Stromal tumor	1	1	0		
NET	1	6	2		
P-J polypus	0	1	1		
Pre-HB	134.9 ± 18.8	129.8 ± 19.1	126.1 ± 30.5	0.23	0.15
Pre-TBIL	14.0 ± 6.8	29.5 ± 43.7	22.3 ± 13.2	0.12	0.44
Pre-DBIL	3.8 ± 2.1	21.6 ± 43.8	17.6 ± 15.8	0.32	0.51
CA19-9	19.1 ± 21.7	30.6 ± 39.9	22.6 ± 9.9	0.18	0.46
CEA	1.92 ± 1.4	4.1 ± 6.8	3.1 ± 1.6	0.27	0.61

LTDA, Laparoscopic transduodenal ampullectomy; LPD, Laparoscopic pancreaticoduodenectomy; OTDA, Open transduodenal ampullectomy; BMI, Body mass index; TVA, Tubulovillous adenoma; NET, Neuroendocrine tumor; CIHGIN, Chronic inflammation with high-grade intraepithelial neoplasia; P-J polypus, Peutz–Jeghers polypus; Pre-HB, Pre-operative hemoglobin; Pre-WBC, Pre-operative white blood cells; Pre-TBIL, Pre-operative total bilirubin; Pre-DBIL, Pre-operative direct bilirubin; CEA, Carcinoembryonic antigen; p^1^-value, the p-value of comparison between group 1 and group 2; p^2^-value, the p-value of comparison between group 1 and group 3.

The operative details and postoperative outcomes are shown in **Tables 2**, **3**, respectively. One patient in group 1 was converted to LPD due to rapid frozen pathological examination indicating a malignant lesion. The patients required neither intraoperative blood transfusions nor the conversion to open surgery. Compared to the patients in group 2, the patients in group 1 required less operative time (159.7 ± 47.5 min vs. 298.1 ± 62.6 min, *p* < 0.01) and suffered from lesser blood loss (23.3 ±16.7 ml vs. 156.8 ±112.1 ml, *p* = 0.002). Compared to the patients in group 3, the patients in group 1 suffered from less blood loss (23.3 ± 16.7 ml vs. 123.7 ± 132.6 ml, *p* = 0.03). The operative time between two groups was comparable. All patients in this study achieved R0 resection. Compared to that in group 2, the postoperative hospital stays (POHS) were significantly shorter for patients in group 1 (9.0 ± 5.3 days vs. 15.5 ± 7.3 days, *p* = 0.04). In terms of complications, one patient in group 1 and two patients in group 3 suffered from delayed gastric emptying, which was managed by conservative therapy. However, more patients in group 2 suffered from postoperative complications, including three cases of clinically relevant pancreatic fistula (grade B: 2 cases, grade C: 1 case), one case of delayed gastric emptying, one case of bile leakage, and one case of an abdominal abscess. One patient in group 1 reported gastrointestinal bleeding after discharge, which was managed by arterial-embolization therapy. One patient in group 2 discharged on POD 9 suffered from abdominal bleeding on the fourth day after discharge and required re-operations. The patient was finally discharged 32 days after the last operation. One patient in group 3 who suffered from abdominal abscess and gastrointestinal bleeding required re-operation. Another patient in group 3 who suffered from abdominal abscess required percutaneous drainage. The 90-day mortality of patients included in this study was 0. All patients included in this study were followed up by outpatient department visits or by telephonic assessment every 6 months and the median follow-up period was 17 months. No patients suffered from the recurrence of tumors during the follow-up period.

**Table 2:** The operative outcomes.

Variables	LTDA	LPD	OTDA	*p* ^1^-value	*p* ^2^-value
No. of patients	9	19	15	–	–
OT (min)	159.7 ± 47.5	298.1 ± 62.6	138.2 ± 36.2	<0.01	0.37
EBL (ml)	23.3 ± 16.7	156.8 ± 112.1	123.7 ± 132.6	0.002	0.03
R0 resection	9, 100%	19, 100%	15, 100%	–	–
Depth of ductal invasion (mm)	1.5 ± 0.3	2.6 ± 0.8	1.5 ± 0.7	0.08	0.88
Conversion to open surgery (*n*, %)	0	0	–		–
Transfusion (*n*, %)	0	0	0	–	–
Tumor size (cm)	1.9 ± 0.5	2.0 ± 0.9	2.3 ± 0.9	0.87	0.65

LTDA, Laparoscopic transduodenal ampullectomy; LPD, Laparoscopic pancreaticoduodenectomy; OTDA, Open transduodenal ampullectomy; OT, Operative time; EBL, Estimated blood loss; p^1^-value, the p-value of comparison between group 1 and group 2; p^2^-value, the p-value of comparison between group 1 and group 3.

**Table 3:** The post-operative outcomes.

Variables	LTDA	LPD	OTDA	*p* ^1^-value	*p* ^2^-value
No. of patients	9	19	15	–	–
Time to oral intake (days)	1.3 ± 1.1	1.6 ± 0.8	2.8 ± 0.9	0.42	0.02
POHS (days)	9.0 ± 5.3	15.5 ± 7.3	9.3 ± 6.9	0.04	0.34
Overall complications (*n*, %)	2, 22.2%	7, 36.8%	6, 40%	0.67	0.66
Clavien–Dindo ≥ III (*n*, %)	1, 11.1%	1, 5.3%	1, 6.7%	1.0	1.0
Re-operation (*n*, %)	0	1, 5.3%	1, 6.7%	–	–
Pancreatic fistula (*n*, %)				0.53	–
Grade B	0	2, 10.6%	0		
Grade C	0	1, 5.3%	0		
Bile leakage (*n*, %)	0	1, 5.3%	0	1.0	–
DGE (*n*, %)	1, 11.1%	1, 5.3%	2, 13.4%	1.0	1.0
Abdominal bleeding (*n*, %)	0	1, 5.3%	0	1.0	–
Abdominal abscess (*n*, %)	0	1, 5.3%	2, 13.4%	1.0	0.51
Gastrointestinal bleeding (*n*, %)	1, 11.1%	0	1, 6.7%	0.32	1.0
90-day mortality	0	0	0	–	–

LTDA, Laparoscopic transduodenal ampullectomy; LPD, Laparoscopic pancreaticoduodenectomy; OTDA, Open transduodenal ampullectomy; POHS, Post-operative hospital stays; DGE, Delayed gastric emptying. p^1^-value, the p-value of comparison between group 1 and group 2; p^2^-value, the p-value of comparison between group 1 and group 3.

## Discussion

The adenoma–carcinoma sequence is believed to be related to the malignant transformation of ampullary tumors, which is validated by histological observation of transitional stages from adenoma with mild, moderate, and severe cellular atypia to invasive carcinoma (10). Therefore, prompt recognition, diagnosis, and removal of these lesions have become the standard of care (11). There are three therapeutic strategies for tumors of AoV, including EP, TDA, and PD. For malignant ampullary tumors, PD is the standard treatment of choice. Apart from removing the primary tumor, PD can provide extensive lymphadenectomy, which is particularly important because nodal status is one of the most significant predictors of survival in patients with carcinoma of AoV (12). In the setting of benign ampullary lesions, EP was found to be equally effective with lower rates of morbidity and identical mortality when compared to surgical ampullectomies (SAs) (13). EP is now considered the gold standard for the treatment of benign tumors of AoV (14). According to the European Society of Gastrointestinal Endoscopy (ESGE) guidelines for ampullary tumors, TDA should be considered when endoscopic resection is not feasible due to technicalities (e.g., diverticulum, size > 4 cm) or in the case of intraductal involvement (of >20 mm) (15). Multidisciplinary preoperative planning should be carried out before conducting the procedure (16).

Accurate preoperative diagnosis and staging of ampullary adenomatous lesions are critical for the prediction of prognosis and the determination of the most suitable therapeutic approach. Gastroscopy and biopsy of the lesion should be routinely performed in all patients. Performing preoperative endoscopic ultrasonography and/or abdominal magnetic resonance cholangiopancreatography (MRCP) is essential to confirm the diagnosis and depth of lesion invasion before performing TDA (15). However, the preoperative diagnostic accuracy is not very high, particularly in the diagnosis of adenoma (17). Sekine et al. reported an 83.3% preoperative diagnostic accuracy rate and stressed the importance of a complete excision biopsy (18). Intraoperative rapid frozen pathological examinations of an intact specimen are critical. All patients who underwent LTDA consented to the possibility of an LPD, with the intraoperative determination based on the frozen section results. One patient in this study was required to be converted to LPD.

Compared to open surgeries, laparoscopic surgeries can have several advantages, including earlier recovery, lower complications, and better cosmetic outcomes. However, it is challenging to perform transduodenal ampullectomy laparoscopically and only a few case reports are available in the literature. To the best of our knowledge, we reported the largest number of LTDA in the literature. The operative outcomes were also more favorable compared to that in the literature.

In open surgeries, the location of AoV can be easily identified by palpating the mass in the duodenal cavity. However, in laparoscopic approaches, the effectiveness of palpation is limited. Generally, the position of AOV is relatively fixed. In this study, the duodenum was opened at the lower third of the descending duodenum, and AoV was successfully located in the first two cases. However, AoV was not found when the duodenum was opened at the same position in the third patient. We were then forced to open the duodenum more widely, and finally, AoV was located at the beginning of the horizontal segment of the duodenum. In the process of searching for AoV, repeated clamping of the duodenal mucosa led to significant swelling of the duodenal mucosa, causing difficulties in performing ampullectomy. Furthermore, due to the duodenal incision being too large, it was also very difficult to close the duodenal incision. Therefore, it is critical to identify the accurate location of AoV. There are several strategies to locate the ampulla. A preoperative endoscopic retrograde biliary drainage catheter could assist in locating the AoV. However, the procedure may cause acute pancreatitis, which could interfere with ampullectomy. To locate the ampulla, Logarajah et al. performed a partial cystic ductotomy and fed a rubber tube through the cystic duct until it entered the duodenum (11). Intraoperative ultrasound is an atraumatic tool that can be used to locate the AoV. The precise opening of the duodenum can also reduce the difficulty of closing the duodenal incision.

It is very important to maintain good exposure and a clean surgical field for laparoscopic surgery. The mucosa of the duodenum is fragile and prone to bleeding. Physiological saline with noradrenaline was routinely injected into the submucosa of AoV, which can aid in lifting the lesion, thereby reducing mucosal bleeding. Ahn et al. did not carry out sphincteroplasty until they removed the tumor completely (6). However, we found that if the tumor was completely removed, the bile duct and pancreatic duct would shrink back into the parenchyma of the pancreas, increasing the difficulty of carrying out sphincteroplasty. Therefore, the pancreatic duct was first opened, and the common bile duct was retained for retraction. In the setting of the common bile duct to duodenum mucosa sphincteroplasty, the common bile duct was opened three-quarters circumferentially, retaining the cephalad common bile duct wall for retraction. Sphincteroplasty could then be carried out safely and speedily with good exposure.

Compared to LPD, the operative outcomes of LTDA were much more favorable, with significantly less operative time and lower blood losses. Patients with pre-malignant ampullary tumors often involve a soft pancreas with a small duct, increasing the risk of pancreatic fistula (19). Therefore, more patients in the PD group suffered from complications. Only one patient suffered from postoperative complications, which were managed by conservative therapy. The postoperative hospital stays were also significantly shorter in the LTDA group due to lower postoperative complications.

Local recurrence after ampullectomy is uncommon, but it does occur, even in benign adenomas. Logarajah et al. reported the development of recurrent adenomas in 2 out of 15 patients (13.3%) after OTDA (11). No patient suffered from tumor recurrence. However, the median follow-up period was only 17 months in this study, and longer follow-ups are required to establish a definite conclusion.

There are several limitations of this study. It is a retrospective study with a small sample size. A prospective, randomized controlled trial (RCT) comparing LTDA to LPD or EP can provide valid pieces of evidence. However, an RCT is difficult to carry out in small numbers of patients undergoing these procedures. Therefore, the present study can substantially contribute to the available evidence despite its limitations.

## Conclusion

In conclusion, LTDA is found to be a safe and feasible procedure in the setting of pre-malignant tumors of AoV in well-selected patients. Preoperative planning at the multidisciplinary level is essential before the surgery. Although these patients require continued follow-up, the benefits of organ preservation may outweigh the requirements of future endoscopic surveillance. However, high-volume, multi-center prospective studies are required to validate the findings of this study and to establish a definite conclusion.

## Data availability statement

The raw data supporting the conclusions of this article will be made available by the authors, without undue reservation.

## Ethics statement

The studies involving human participants were reviewed and approved by Ethics Committee West China Hospital of Sichuan University. The patients/participants provided their written informed consent to participate in this study.

## Author contributions

PG, HC, ZW, BP, and YC designed of the work; PG, HC, ZW and BP collected and analyzed the data for the work; PG, HC and ZW drafted the manuscript; BP and YC revised the manuscript. All authors published and agreed to be accountable for all aspects of the work. All authors approved the final manuscript.
